# Landiolol for rate control in decompensated heart failure due to atrial fibrillation: the LARISA trial

**DOI:** 10.1093/ehjacc/zuag027

**Published:** 2026-02-27

**Authors:** Marek Sramko, Ales Benak, Jakub Toman, Martin Kleissner, Michal Pazdernik, Josef Kautzner

**Affiliations:** Cardiology Department, Institute for Clinical and Experimental Medicine (IKEM), Videnska 1958/9, Prague 14021, Czech Republic; 2nd Department of Cardiology and Angiology, General University Hospital, Prague, Czech Republic; Cardiology Department, Institute for Clinical and Experimental Medicine (IKEM), Videnska 1958/9, Prague 14021, Czech Republic; Cardiology Department, Institute for Clinical and Experimental Medicine (IKEM), Videnska 1958/9, Prague 14021, Czech Republic; Cardiology Department, Institute for Clinical and Experimental Medicine (IKEM), Videnska 1958/9, Prague 14021, Czech Republic; Cardiology Department, Institute for Clinical and Experimental Medicine (IKEM), Videnska 1958/9, Prague 14021, Czech Republic; Cardiology Department, Institute for Clinical and Experimental Medicine (IKEM), Videnska 1958/9, Prague 14021, Czech Republic

**Keywords:** Heart failure therapy, Arrhythmia, Rate control, Haemodynamics

## Abstract

**Aims:**

Rate control of atrial fibrillation (AF) in acute heart failure (AHF) with reduced left ventricular ejection fraction (LVEF) is challenging. We evaluated the safety and efficacy of intensive rate control (IRC) using landiolol with adjunctive digoxin.

**Methods and results:**

Forty patients with AHF due to AF [heart rate (HR) > 130/min, LVEF <40%] were randomized to IRC with landiolol plus digoxin or standard rate control therapy. The primary endpoint was sustained HR reduction >20% or <115/min at 2 h. Haemodynamics, dyspnoea, and lung congestion were assessed serially. HR control was achieved faster in the IRC vs. standard group (median 1.5 vs. 4 h; *P* = 0.016). At 2 h, HR decreased by 40% of the IRC group vs. 23% of standard therapy group (*P* = 0.045), and the primary endpoint was reached in 80% vs. 20% of the patients, respectively (*P* = 0.04). IRC resulted in greater dyspnoea relief and lung decongestion without excess hypotension or adverse events. At 24 h, outcomes between the groups were similar.

**Conclusion:**

In AHF with reduced LVEF, protocolized landiolol-based intensive rate control provides faster and safe HR reduction with early symptomatic benefit compared with standard therapy.

**Pre-registered Clinical Trial Number:**  ClinicalTrials.org: NCT04694092.

Key points

- In patients with acute heart failure and reduced left ventricular ejection fraction, intensive rate control with landiolol plus digoxin achieved significantly faster and more effective heart rate reduction than standard therapy.
- Rapid protocolized titration of landiolol improved dyspnoea and lung congestion without causing sustained hypotension or treatment-related adverse events, demonstrating a favourable short-term safety profile.
- Despite early advantages, clinical and haemodynamic outcomes at 24 h were similar between strategies, indicating that the strategy of intensive rate control by landiolol primarily provides early stabilization and symptomatic benefit.

## Introduction

Acute rate control of atrial fibrillation (AF) in patients with acute heart failure (AHF) and reduced left ventricular ejection fraction (LVEF) remains challenging, as most rate-controlling drugs exert negative inotropic effects.^1,2^ Evidence supporting specific, protocolized rate-control strategies in this setting is limited, and current guidelines provide little guidance regarding the optimal choice of agent, dosing, and speed of titration in the hyperacute phase.^1,2^ Landiolol is a short-acting intravenous beta blocker with mitigated negative inotropy and a favourable safety profile.^3,4^ Accordingly, this study evaluated the safety and efficacy of a protocolized intensive rate-control (IRC) strategy based on landiolol with adjunctive digoxin in patients with AHF and reduced LVEF.

## Methods

Patients with AHF caused by AF with heart rate (HR) > 130/min and LVEF <40% were randomized to either IRC with landiolol and adjunctive digoxin vs. standard rate control therapy. Excluded were secondary causes of AF and patients who received non-chronic rate control drugs <24 h before the study. The primary endpoint was a sustained decrease of HR by >20% or below 115/min at 2 h.

Both groups received AHF therapy as needed.^1^ In addition, for the first 2 h, the patients in the standard group could receive any guideline-recommended rate control therapy, except for landiolol^2^; whereas the patients in the IRC group could receive only landiolol and boluses of digoxin (a maximum of 0.5 mg/2 h). The choice of the rate control drugs in the standard therapy was left on the clinical judgement of independent clinicians. Landiolol was started at 10 µg/kg/min, and the dose was increased by 10 µg/kg/min every 10–15 min until reaching 80 µg/kg/min, achieving the primary endpoint, or development of hypotension [systolic blood pressure (SBP) < 80 mmHg or mean arterial pressure < map > < 60 mmHg], whichever first. In case of hypotension, the infusion rate was immediately lowered to the previous dose. After 2 h, both groups were allowed to receive any approved rate control therapy, although the default strategy was to restore sinus rhythm by electric cardioversion whenever it was safe.

Blood pressure was monitored continuously through a radial artery cannula. Haemodynamics, laboratory parameters, lung congestion (total count of B-lines on lung ultrasound), and subjective dyspnoea (visual analogue score 1–10) were evaluated at baseline, 2, 12, and 24 h. Groups were compared by *t*-test, Wilcoxon test, chi-square test, and Fisher’s test. Within-subject changes were evaluated by two-tailed paired *t*-tests. The study was pre-registered on ClinicalTrials.org (NCT04694092) and approved by the institutional ethics committee. All patients signed informed consent.

## Results

A total of 40 patients were included (*Table 1*). The typical clinical presentation of AHF was pulmonary oedema with orthopnoea, mean HR of 154/min, SBP of 129 mmHg, B-type natriuretic peptide (BNP) of 965 ng/L, and arterial lactate of 2.0 mmol/L (*Table 2*). In most patients, AF presented *de novo* (73%) and lasted for >48 h (83%). Only 32% of the patients were on chronic anticoagulation therapy, and 47% were on beta blockers for heart failure or hypertension (metoprolol equivalent of 82 mg/day). Left atrial thrombus was present in 7 of 32 patients (22%) who underwent transoesophageal echocardiography. Patients in the IRC group (*n* = 20) vs. standard group (*n* = 20) were older (71 vs. 64 years, *P* = 0.011) and had more prevalent diabetes (55% vs. 20%, *P* = 0.048), but they did not differ in any other baseline characteristics (*Table 1*).

**Table 1:** Baseline clinical characteristics

	Total (*n* = 40)	Intensive rate control (*n* = 20)	Standard therapy (*n* = 20)	*P*-value
Age (years)	67 ± 10	71 ± 8	64 ± 10	0.011*
Male gender	21 (53)	8 (40)	13 (65)	0.2
Body mass index (kg/m^2^)	33 ± 7	33 ± 7	32 ± 7	0.8
Hypertension	28 (70)	16 (80)	12 (60)	0.3
Diabetes mellitus	15 (38)	11 (55)	4 (20)	0.048*
Previous heart failure	9 (23)	5 (25)	4 (20)	1.0
Coronary artery disease	6 (15)	3 (15)	3 (15)	1.0
*De novo* AF	29 (73)	15 (75)	14 (70)	0.8
AF duration >48 h or unknown	32 (80)	17 (85)	15 (75)	0.7
**Chronic medication**				
Anticoagulation	13 (33)	6 (30)	7 (35)	1.0
ACEI/ARB/ARNI	21 (53)	11 (55)	10 (50)	1.0
Beta blocker	19 (48)	10 (50)	9 (45)	1.0
Beta blocker daily dose (% of maximum recommended dose)	41 ± 33	36 ± 38	48 ± 32	0.8
Digoxin	2 (5)	1 (5)	1 (5)	1.0
Loop diuretics	8 (20)	4 (20)	4 (20)	1.0
Glyphosine	1 (2.5)	0 (0.0)	1 (5)	1.0
**Echocardiography**				
LV EDD index (mm/m)	32 ± 5	31 ± 4	33 ± 5	0.1
LV ejection fraction (%)	33 ± 11	32 ± 9	33 ± 12	0.8
LV ejection fraction ≤35%	14 (35)	7 (35)	7 (35)	1.0
Left atrial diameter (mm)	47 ± 6	46 ± 6	49 ± 7	0.2
MR grade (1 = mild, 2 = moderate, 3 = moderate/severe, 4 = severe)	2.0 ± 1.0	2.3 ± 1.1	1.8 ± 0.9	0.1
RV moderate/severe dysfunction	24 (60)	12 (60)	12 (60)	1.0
LAA thrombus^a^	7/32 (22)	3/15 (20)	4/17 (24)	1.0

The values are mean ± and count (percentage). ACEI, angiotensin-converting enzyme inhibitor; AF, atrial fibrillation; ARB, angiotensin receptor blocker; ARNI, angiotensin receptor-neprilysin inhibitor; COPD, chronic obstructive pulmonary disease; EDD, end-diastolic diameter; LAA, left atrial appendage; LV, left ventricle; MR, mitral regurgitation; RV, right ventricle; TR, tricuspid regurgitation.

*P < 0.05.

^a^Calculated in patients who underwent transoesophageal echocardiography.

**Table 2:** Haemodynamics

	Baseline		2 h		12 h		24 h	
	Standard (*n* = 20)	Intensive (*n* = 20)	Standard (*n* = 20)	Intensive (*n* = 20)	Standard (*n* = 20)	Intensive (*n* = 20)	Standard (*n* = 18)	Intensive (*n* = 20)
Sinus rhythm	0 (0)	0 (0)	0 (0)	0 (0)	9 (45)	3 (15)	9 (50)	10 (50)
HR	153 (18)	156 (19)	129 (23)	117 (23)*	94 (19)	109 (28)	94 (24)	93 (23)
HR <115/min	0 (0)	0 (0)	5 (25)	11 (55)*	18 (90)	12 (60)	15 (83)	17 (85)
HR decrease >20%	NA	NA	7 (35)	14 (70)*	18 (90)	14 (70)	17 (94)	17 (85)
Achieved rate control^a^	0 (0)	0 (0)	7 (35)	16 (80)**	18 (90)	15 (75)	17 (94)	17 (85)
SBP (mmHg)	131 (18)	126 (22)	129 (24)	116 (20)*	122 (22)	122 (21)	118 (12)	121 (19)
MAP (mmHg)	99 (13)	95 (16)	94 (12)	85 (13)**	87 (13)	88 (16)	85 (10)	85 (13)
Hypotension^b^	0 (0)	1 (5)	1 (5)	0 (0)	1 (5)	(0)	1 (5)	(0)
Congestion at CXR	19 (95)	17 (85)	NA	NA	NA	NA	7 (39%)	4 (20%)
B-lines total count (LUS)	17 (13)	20 (10)	12 (12)	12 (7)	8 (6)	9 (8)	7 (7)	8 (8)
Symptoms score (1–10)	5.5 (2.0)	6.2 (1.7)	4.0 (1.7)	3.2 (1.5)	2.5 (1.6)	3.0 (1.7)	2.8 (1.8)	2.7 (1.9)
Total diuresis (ml)	NA	NA	1089 (541)	809 (505)	NA	NA	3090 (1452)	3130 (1010)
Lactate (mmol/L)	1.7 (0.9)	2.6 (2.8)	1.3 (0.4)	2.1 (2.2)	1.0 (0.3)	1.4 (0.7)	1.2 (0.3)	1.3 (0.4)
BNP (ng/L)	786 (302; 1080)	827 (468; 1801)	NA	NA	806 (411; 1518)	940 (650; 1145)	734 (393; 1083)	786 (516; 965)

BNP, B-type natriuretic peptide; CXR, chest X-ray; HR, heart rate; LUS, lung ultrasound; MAP, mean arterial pressure; SBP, systolic blood pressure.

*/**P-value of 0.05–0.1/ < 0.05 for between-group comparisons at the respective time.

^a^HR <115/min or HR decrease >20% from baseline.

^b^SBP <85 mmHg or MAP <65 mmHg or vasopressors to maintain blood pressure above the values.

**Figure 1:**
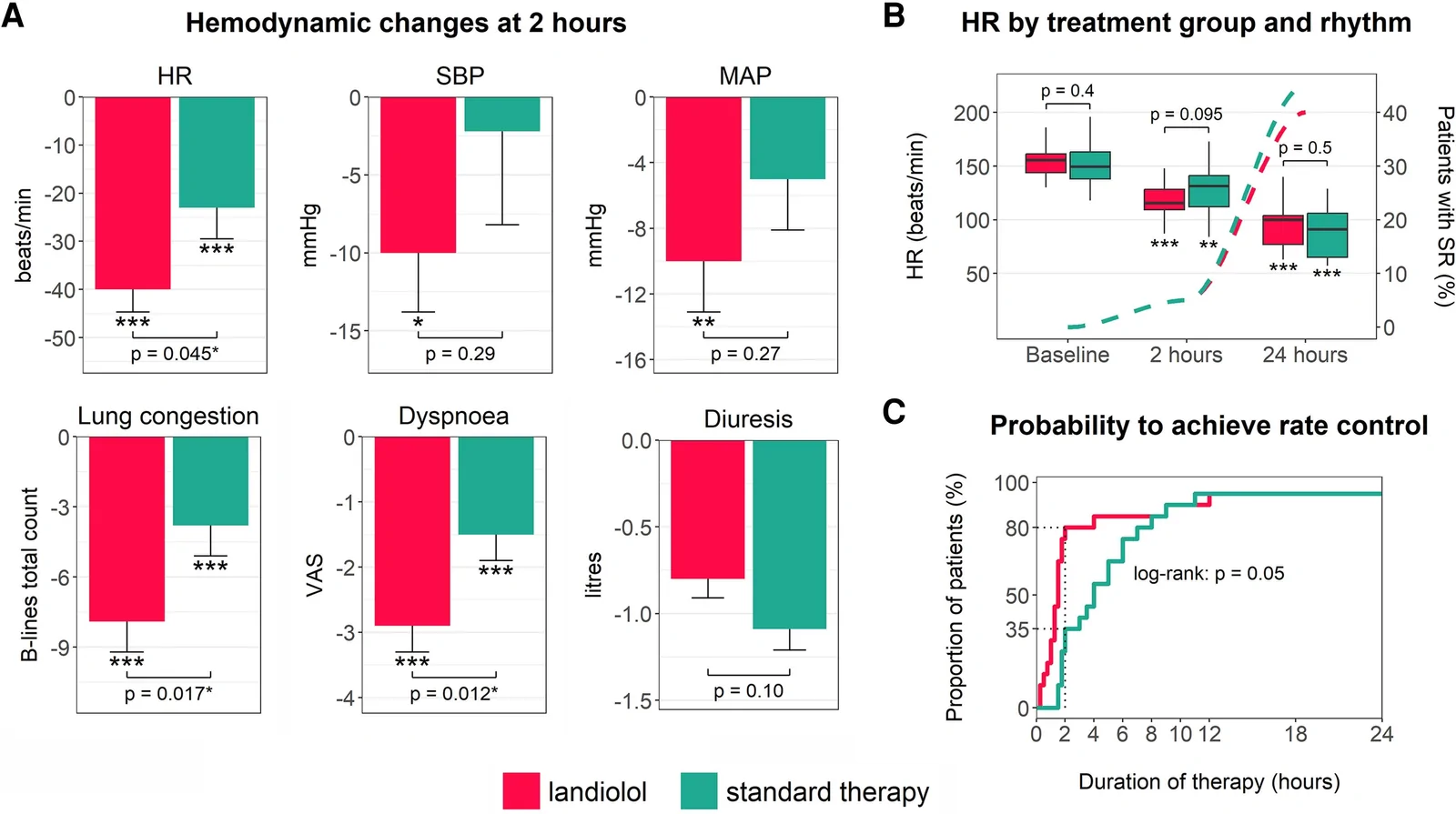
**Haemodynamic changes.** The *P*-values represent between-group comparison of the changes; *P < 0.05, **P = 0.01, and ***P = 0.001 for within-group changes from baseline. Panel (*A*) changes of the hemodynamic parameters at 2 h compared to baseline, (*B*) comparison of heart rate at baseline, 2 h and 24 h with regard to the present sinus rhythm, (*C*) analysis of the time to achieve rate control.

Besides the type of beta blockers, the therapy during the first 2 h did not differ between the IRC vs. standard group [landiolol: 100% vs. 0%, other beta blockers than landiolol: 0% vs. 35% (metoprolol equivalent dose of 19 mg), amiodarone: 0 vs. 15%, digoxin: 0.39 vs. 0.38 mg, nitrates: 20% vs. 20%, inotropes: 5% vs. 5%, furosemide: 85 vs. 60 mg, non-invasive ventilation: 5% vs. 5%, vasopressors: none]. The average titrated dose of landiolol was 45 ± 27 µg/kg/min, and the maximum dose of 80 µg/kg/min was reached in 6 (30%) of the patients. After 2 h of therapy, HR decreased on average by 40% in the IRC group vs. 23% in the standard group (*P* = 0.045), and the primary endpoint was reached in 80% vs. 20% of the patients, respectively (*P* = 0.04, *Figure 1*). The primary endpoint was achieved during a median of 1.5 h [interquartile range (IQR) 1–2] in the IRC group vs. 4 h (IQR 2–6) in the standard group (*P* = 0.016). Compared with the standard group, patients in the IRC group had greater improvement of dyspnoea score and more prominent lung decongestion, despite similar cumulative diuresis (*Figure 1*). There was a greater decrease in blood pressure in the IRC group (*Figure 1*), but no patient in either group developed sustained hypotension, progression of AHF, or any other therapy-related adverse events.

On average, the landiolol infusion was administered for 5 h, and the cumulative dose reached 525 mg. After 24 h, the intensive rate control vs. standard group did not differ in the use of non-landiolol beta blockers (75% vs. 70%), amiodarone (25% vs. 30%), performed electric cardioversion (50% and 45%), persistence of sinus rhythm (50% and 50%), HR (93 vs. 94 beats/min), achieved primary endpoint (85% vs. 94%), SBP (121 vs. 118 mmHg), dyspnoea score (2.7 vs. 2.8), B-lines count (8 vs. 7), cumulative diuresis (3130 vs. 3090 mL), BNP (786 vs. 734, ng/L), or arterial lactate (1.3 vs. 1.2 mmol/L; all *P* > 0.2).

## Discussion

Acute management of AF in patients with AHF and reduced LVEF remains challenging, particularly when immediate cardioversion is unfeasible and rate control becomes the primary strategy. While contemporary guidelines emphasize ventricular rate control, they acknowledge the limited evidence guiding drug selection and titration in the acute phase.^1,2^ Our study demonstrated that a protocolized strategy of IRC using landiolol with adjunctive digoxin achieved faster heart-rate reduction and earlier symptomatic improvement compared with standard therapy. The short-acting and β_1_-selective pharmacological profile of landiolol allowed precise titration under close haemodynamic monitoring and was likely responsible for the absence of hypotension or adverse events. The achieved rate control and haemodynamic stabilization created favourable conditions for subsequent rhythm-control interventions.

Various rhythm-control strategies have been shown to improve long-term outcomes in selected patients with chronic heart failure.^5,6^ The applicability of these studies to the hyperacute AHF setting is limited, where immediate stabilization rather than long-term rhythm maintenance is the primary therapeutic goal. In this regard, our study provides original evidence that can have implications for clinical practice. These findings corroborate prior studies in Asian populations demonstrating the efficacy of landiolol for acute rate control of AF in patients with or without AHF.^7,8^

This was a pilot study limited by its non-blinded design and non-protocolized standard therapy, which nevertheless reflected contemporary clinical practice. Most patients presented with normotensive pulmonary oedema without cardiogenic shock. Further research is warranted to evaluate the safety of landiolol in more severe heart failure. Additional studies are also needed to clarify the optimal dosing of landiolol in Caucasian patient populations, as required doses appear to be higher than those reported in Asian populations.^7,8^

## Data Availability

The data underlying this article will be shared on reasonable request to the corresponding author.
